# Histological and molecular analysis of a progressive diffuse intrinsic pontine glioma and synchronous metastatic lesions: a case report

**DOI:** 10.18632/oncotarget.10034

**Published:** 2016-06-14

**Authors:** Javad Nazarian, Gary E. Mason, Cheng Ying Ho, Eshini Panditharatna, Madhuri Kambhampati, L. Gilbert Vezina, Roger J. Packer, Eugene I. Hwang

**Affiliations:** ^1^ Center for Genetic Medicine Research, Children's National Medical Center, Washington, DC, USA; ^2^ Department of Integrative Systems Biology, George Washington University School of Medicine and Health Sciences, Washington, DC, USA; ^3^ University of Pittsburgh School of Medicine, Pittsburgh, PA, USA; ^4^ Department of Pathology, Children's National Medical Center, Washington, DC, USA; ^5^ Division of Neuro-radiology, Children's National Medical Center, Washington, DC, USA; ^6^ Institute for Biomedical Sciences, George Washington University School of Medicine and Health Sciences, Washington, DC, USA; ^7^ Brain Tumor Institute, Daniel and Jennifer Gilbert Neurofibromatosis Institute, Neuroscience and Behavioral Medicine, Children's National Medical Center, NW, Washington, DC, USA; ^8^ Center for Cancer and Blood Disorders, Children's National Medical Center, Washington, DC, USA

**Keywords:** DIPG, metastasis, molecular, phenotype, autopsy tissue, Pathology Section

## Abstract

There is no curative treatment for patients with diffuse intrinsic pontine glioma (DIPG). However, with the recent availability of biopsy and autopsy tissue, new data regarding the biologic behavior of this tumor have emerged, allowing greater molecular characterization and leading to investigations which may result in improved therapeutic options. Treatment strategies must address both primary disease sites as well as any metastatic deposits, which may be variably sensitive to a particular approach.

In this case report, we present a patient with DIPG treated with irradiation and serial investigational agents. The clinical, pathological and molecular phenotypes of both the progressive primary tumor as well as concomitant metastatic deposits obtained at autopsy are discussed. While some mRNA differences were demonstrated, all analyzed sites of disease shared similar mutational arrangements, suggesting that targeting the mutations of the primary tumor may be effective for all sites of disease.

## INTRODUCTION

Diffuse intrinsic pontine glioma (DIPG) is a subset of pediatric brainstem glioma with an abysmal median survival of 10-12 months despite multiple clinical trials testing myriad new treatments.[1] The majority of DIPG tumors progress locally but metastases can occur.[2, 3] Due to the rarity of available tissue, most studies have not analyzed the molecular and pathological differences between primary and metastatic disease. These differences may inform our conceptualization of DIPG and lead to improvement in treatments. We describe a case of progressive disease at both the primary and two new metastatic points, and present the molecular and pathological features of the primary and metastatic tumor.

## CASE PRESENTATION

A previously healthy 9-year-old female presented to her primary care physician after developing left-sided facial weakness. She was referred for magnetic resonance imaging (MRI) which showed T2/FLAIR hyperintensity centered within and expanding the pons. The initial physical exam revealed several neurological abnormalities including a left 6^th^ nerve palsy with bilateral nystagmus as well as an incomplete left facial palsy and left-sided dysmetria.

### Treatments administered

**Figure 1:**
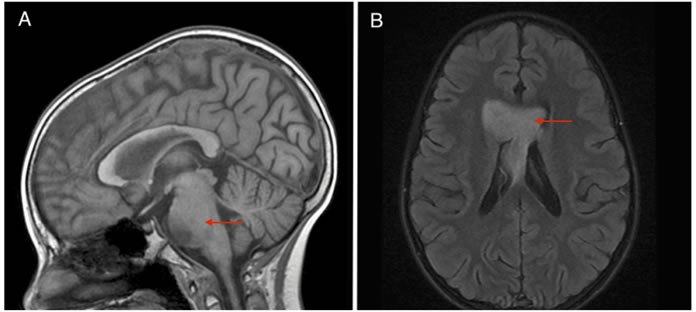
**A.** Sagittal SPGR of brainstem tumor progression and **B.** Axial T2 FLAIR contrasted images of concomitant metastatic lesion growth

The patient was enrolled on a Children's Oncology Group trial with vorinostat and focal radiation therapy; the post-radiation MRI revealed improvement in the pontine lesion with decreased mass effect. However, seven months later the pontine glioma increased in size and two new metastases were simultaneously noted: 1) a large lesion of the septum pellucidum involving the frontal horns of the lateral ventricles and the undersurface of the anterior corpus callosum (labeled ‘SP’ metastatic point), and 2) a left posterior hippocampal lesion (labeled ‘PH’ lesion) (Figure 1A, 1B).

The patient was next enrolled in a Pediatric Brain Tumor Consortium trial and received two doses of a telomerase inhibitor which was discontinued for reasons unrelated to the patient's clinical course, and she was noted to have subsequent progression of both the primary and metastatic lesions. After the patient died, an autopsy was performed within 10 hours of death, and fresh-frozen and formalin fixed tissue was obtained from the primary site and the disseminated lesions as well as the grossly normal brain.

### Tissue analysis

Coronal sections of the post-mortem brain showed the SP tumor: a large, ill-defined metastatic solid mass centered at the septum pellucidum and involving the corpus callosum, right internal capsule, and the frontal horns of the lateral ventricles. Autopsy also revealed the PH lesion: a smaller lesion centered in the left posterior hippocampus. The basis pontis was significantly expanded, while the cerebellum was grossly normal.

**Figure 2:**
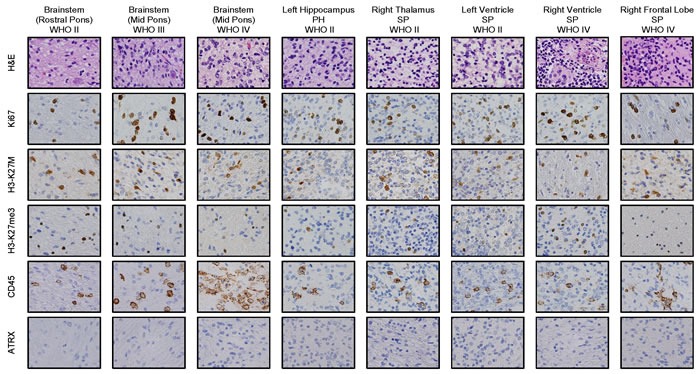
Immunostaining of primary site of tumor and tumor metastases to left hippocampus (WHO grade II, PH lesion), and SP tumor: right thalamus (WHO grade II), left ventricle (WHO grade II), right ventricle (WHO grade IV) and right frontal lobe (WHO grade IV) show histone 3 K27M (H3K27M) mutation and decreased H3K27 trimethylation (H3K27me3) Tumorigenesis is indicated by hypercellularity and proliferative nature indicated by H&E (a) and Ki67 (b) stains, respectively. The positive immunostaining in tumor regions (c, d) represents H3K27M mutant antigen (c) and H3K27me3 status (d). The positive stain of CD45 shows the microglial infiltration (e). ATRX staining is negative in all the tumor types (f).

Histological analysis was undertaken of multiple sites in both the primary and the two metastatic tumor sites, showing that the SP tumor, much like the primary site, was overall best classified as high-grade (WHO Grade IV, Figure 2) while the PH tumor was lower grade; nevertheless, all sites of disease displayed focal necrosis and vascular proliferation. The metastatic tumors had scattered areas of small, round blue cells reminiscent of PNET, but were strongly diffusely positive for GFAP, negative for synaptophysin and were overall best classified as glioma. Metastatic disease in the SP tumor had an increased Ki67 proliferation index compared to the brainstem lesion, 30% *vs*. 8%, respectively. All tumor sites displayed positive staining for the histone 3 K27M (H3K27M) mutation with less evident histological staining for wild type histone 3 trimethylation (H3K27me3) (Figure 2). The tumors were also positive for CD45 representing various degrees of infiltrating resident microglia and macrophages (Figure 2).

**Figure 3:**
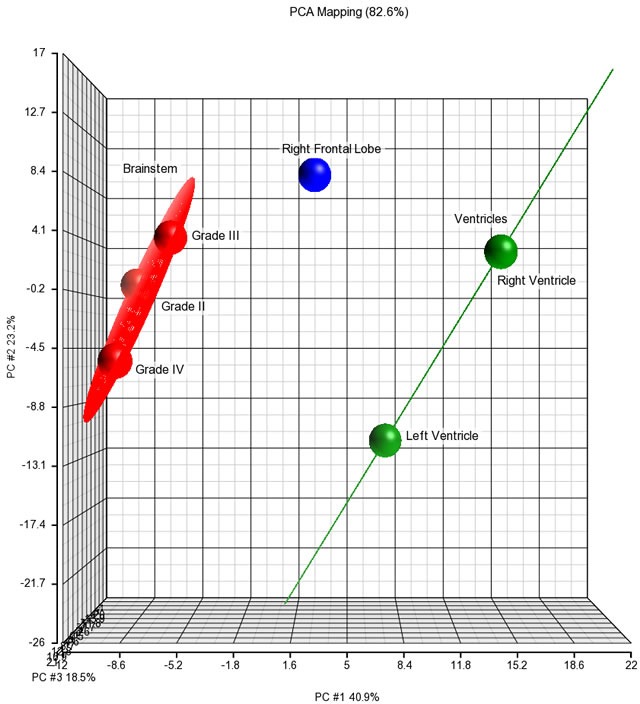
Principal component analysis of differentially expressed mRNA species in brainstem, metastatic right frontal lobe, and metastatic lateral ventricular tumor samples

We extracted mRNA from six brain locations to attempt to differentiate metastatic from primary tumor based on molecular signature, including three locations from the brainstem tumor and 3 sites from the SP tumor (right and left ventricular portions as well as the right frontal portion). No mRNA could be extracted from the PH lesion due to fixation issues. mRNA profiling was completed using the NanoString platform (Cancer Panel) and the differential mRNA expression pattern between the primary brainstem and metastatic tumors was assessed using Partek Genomic Suite software. mRNA profiles of different sites of the large SP tumor were variably similar to the brainstem tumor (Figure 3). Ingenuity pathway analysis revealed that p53 signaling, cell cycle regulation, DNA damage response, growth arrest and DNA damage-inducible 45 (GADD45), and ATM signaling were common tumorigenic pathways between primary and all sites of the SP tumor. We identified differentially expressed mRNA species between the primary tumor compared to the ventricular portion (twenty-two species) or the frontal portion (eight species) of the metastatic SP tumor (fold change > 1.5; < -1.5; p < 0.05) (Table 1). For example, expression of the fibroblast growth factor receptor, FGFR3, is up regulated in the brainstem compared to metastatic tumor samples. However, the overall mRNA profiles of these differentially expressed genes primarily exhibit dysfunction of cell cycle regulatory pathways (Table 1). Given the complexities of histological staining for tumor assessment, these identified mRNA profiles may have clinical relevance in rapid identification of potential therapeutic targets.

**Table 1:** Genes exhibit significant differential expression (ANOVA analysis) between the primary brainstem tumor and separate portions of the SP metastatic tumor, as well as between the major portions of the SP tumor (ventricular portion versus frontal lobe portion)

Brainstem *vs*. Ventricular Portion (SP)		
**Gene**	**Fold Change**	***p*-value**
CCND2	2.71	8.89E-03
FGFR3	2.36	1.99E-02
MLH1	−1.53	1.79E-02
ERBB4	−1.68	1.73E-02
AR	−1.82	1.31E-02
TP53	−2.07	2.54E-02
PTPRG	−2.30	7.04E-03
PML	−2.59	6.30E-03
NPM1	−2.71	1.95E-02
BRCA1	−2.80	1.07E-02
PTK7	−2.81	9.22E-03
KIT	−2.98	2.38E-03
MYCN	−3.20	1.53E-02
LMO1	−4.12	1.23E-02
E2F3	−4.77	4.42E-06
BRCA2	−4.87	7.62E-05
GATA1	−5.29	2.38E-02
RAD54L	−5.55	6.68E-03
CDC25C	−6.30	4.02E-05
MYB	−6.97	1.02E-02
WT1	−10.96	1.23E-02
WEE1	−12.09	8.82E-03
**Brainstem *vs*. Right Frontal Lobe (SP)**		
**Gene**	**Fold Change**	***p*-value**
FGFR3	3.37	2.19E-02
CCND2	2.64	1.79E-02
IFNGR1	1.46	1.05E-03
BRCA2	−1.55	4.04E-02
E2F3	−1.92	6.08E-04
PML	−1.99	4.24E-02
CDC25C	−3.05	1.38E-03
GATA1	−7.64	1.38E-02
**Ventricular Portion (SP) *vs*. Right Frontal Lobe (SP)**		
**Gene**	**Fold Change**	***p*-value**
LMO1	8.60	1.81E-02
TP53	4.52	1.86E-02
WEE1	3.63	3.69E-02
WT1	3.30	5.32E-02
BRCA2	3.14	2.91E-04
MYB	2.78	4.82E-02
E2F3	2.49	2.46E-05
RAD54	2.35	3.91E-02
BRCA1	2.17	3.77E-02
MLH1	2.09	1.32E-02
CDC25C	2.07	4.17E-04
KIT	1.75	1.90E-02
AR	1.61	4.54E-02
PTPRG	1.55	5.38E-02
IFNGR1	1.44	1.45E-03

## DISCUSSION

DIPG continues to have a poor prognosis. Focal radiation therapy remains the primary treatment modality and despite multiple clinical trials, chemotherapy has yet to significantly improve overall survival due to both local progression and metastatic recurrence.[1, 4] Because our patient had unusual synchronous growth of both primary and metastatic lesions, this case offered the rare opportunity to compare the histopathological and molecular characteristics of both primary and metastatic tumors at similar biological time points, which may offer insight in constructing optimal treatment strategies.

Recently, advances have been made in understanding the molecular phenotype of DIPG. While DIPG histology can be heterogeneous, ranging from lower-grade astrocytoma to higher-grade astrocytoma,[5] H3.3 K27M mutations have been well described in over two-thirds of DIPGs[2] and may portend a worse overall survival.[5, 6] PDGFRA, MYC and PVT1 amplifications, ATRX, ACVR1 and TP53 mutations and alternative lengthening of telomeres have also been associated with DIPG.[5, 7, 8] Also, a higher incidence of metastases in DIPG than previously appreciated has been described in recent publications, although our case is the first report of comprehensive phenotypic analysis comparing multiple sites in both primary and distant tumor.[2, 5]

In this case, despite histopathological variability among the primary and metastatic sites, all were positive for the H3K27M mutation, highlighting the possible discrepancy between histologic appearance and mutational status. The retention of the same mutational status in multiple metastatic sites is particularly important, as therapy designed to target a particular genetic phenotype must be able to rely on conservation of the same mutation in all sites of disease. Conversely, mRNA analyses clearly identified a small cohort of genes differentiating the primary tumor from different portions of the metastatic SP tumor. These data are at an mRNA level and are based on limited probe sets (NanoString); thus, further validation using genomics and proteomics is warranted. Overall, mRNA expression and pathway analysis indicate de-regulation of cell cycle regulatory pathways. Differences in site-specific mRNA expression may impact effective combinations of therapy that target specific biological pathways, although therapy designed to target epigenetic and cell cycle regulatory mechanisms may be applicable to all sites of disease. Further, the significant differences in mRNA expression even from different locations of the same tumor implies that single biopsy analysis for mRNA expression may be misleading in designing comprehensive targeted treatment plans.

The advent of groundbreaking scientific discoveries in DIPG will continue to rely on the study of tumor tissue at diagnosis and after intervention. This case displays the potential for therapies to address both primary and metastatic tumors, but further validation of the mutational synchrony between disease sites is necessary before reaching broadly applicable conclusions.

### Ethics statement

Investigation has been conducted in accordance with the ethical standards and according to the Declaration of Helsinki and according to national and international guidelines and has been approved by the authors' institutional review board.
