# Addendum: Histological and molecular analysis of a progressive diffuse intrinsic pontine glioma and synchronous metastatic lesions: a case report

**DOI:** 10.18632/oncotarget.28286

**Published:** 2023-05-12

**Authors:** Javad Nazarian, Gary E. Mason, Cheng Ying Ho, Eshini Panditharatna, Madhuri Kambhampati, L. Gilbert Vezina, Roger J. Packer, Eugene I. Hwang

**Affiliations:** ^1^Center for Genetic Medicine Research, Children’s National Medical Center, Washington, DC, USA; ^2^Department of Integrative Systems Biology, George Washington University School of Medicine and Health Sciences, Washington, DC, USA; ^3^University of Pittsburgh School of Medicine, Pittsburgh, PA, USA; ^4^Department of Pathology, Children’s National Medical Center, Washington, DC, USA; ^5^Division of Neuro-radiology, Children’s National Medical Center, Washington, DC, USA; ^6^Institute for Biomedical Sciences, George Washington University School of Medicine and Health Sciences, Washington, DC, USA; ^7^Brain Tumor Institute, Daniel and Jennifer Gilbert Neurofibromatosis Institute, Neuroscience and Behavioral Medicine, Children’s National Medical Center, NW, Washington, DC, USA; ^8^Center for Cancer and Blood Disorders, Children’s National Medical Center, Washington, DC, USA

**This article has an addendum:** Because this was an autopsy-based analysis, autopsy/donation consent was obtained.

Original article: Oncotarget. 2016; 7:42837–42842. 42837-42842. https://doi.org/10.18632/oncotarget.10034

